# Developmental Coordination Disorder in Preschool-Aged Children: A Neuropsychological Perspective on Visuospatial Working Memory and Attentional, Planning, and Decision-Making Processing in Relation to Fundamental Movement Skills

**DOI:** 10.3390/children12091118

**Published:** 2025-08-25

**Authors:** Katerina Asonitou, Dimitra Koutsouki, Thomas Kourtessis, Antonis Kambas

**Affiliations:** 1 Laboratory of Adapted Physical Activity/Developmental and Physical Disabilities, School of Physical Education and Sport Science, National and Kapodistrian University of Athens, 17237 Athens, Greece; dkoutsou@phed.uoa.gr; 2 School of Social Sciences, Department of Early Childhood Education & Care, International Hellenic University, 57400 Thessaloniki, Greece; thkourte@ecec.ihu.gr; 3 Department of Physical Education and Sport Science, Democritus University of Thrace, 69100 Komotini, Greece; akampas@phyed.duth.gr

**Keywords:** developmental coordination disorder (DCD), executive functioning, visuospatial working memory, attention, planning, decision-making, neuropsychology, motor skills, intervention, early childhood

## Abstract

**Highlights:**

**What are the main findings?**

- Preschool children with DCD show significant deficits in attention, visuospatial processing, and planning compared to typically developing peers.
- Receptive attention and simultaneous processing scores accurately classified DCD status in over 70% of cases.

**What is the implication of the main finding?**

- Cognitive profiling using PASS theory can enhance early diagnosis and guide targeted motor–cognitive interventions.
- Strength-based, personalized programs integrating executive function and motor training may improve developmental outcomes in children with DCD.

**Abstract:**

**Background/Objectives**: This study investigates specific neuropsychological functions—visuospatial working memory, attention, planning, and decision-making—among preschool-aged children with Developmental Coordination Disorder (DCD) compared to typically developing (TD) peers. The objective was to assess deficits in selective, sustained, and focused expressive attention, visuospatial and visual/verbal working memory, and decision-making abilities, and to examine their relationship with fundamental motor skills. **Methods**: A comparative study was conducted with Greek preschool-aged children using the Cognitive Assessment System (CAS) to evaluate cognitive processing (planning, attention, simultaneous processing) and the Movement Assessment Battery for Children—Second Edition (MABC-2) to assess motor skills. **Results**: Significant performance disparities were identified between DCD and TD children across attentional and planning domains, with notable cognitive–motor correlations. Discriminant function analyses revealed high classification accuracy (e.g., 73.9% for receptive attention, 79.5% for simultaneous processing), reinforcing the diagnostic value of targeted cognitive indices. Notably, approximately one-third of the children with DCD (17 out of 44) exhibited deficits in one or more cognitive domains. **Conclusions**: These findings highlight the importance of neuropsychological profiling in understanding DCD and suggest that targeted motor interventions may simultaneously enhance executive function. A strength-based, holistic assessment approach is recommended, supported by educator training and evidence-based therapeutic programming.

## 1. Introduction

### 1.1. Developmental Characteristics and Cognitive Associations of DCD

Motor coordination difficulties in early childhood often reflect underlying cognitive processing challenges, particularly in attentional control, planning, and visuospatial reasoning. These capacities are foundational to adaptive behavior and learning, influencing academic skills, motor competence, and everyday functioning [1,2].

A developmental perspective underscores the tight coupling between motor and cognitive domains. For instance, gains in preschoolers’ object control abilities correlate with enhancements in executive functions such as working memory and inhibition [3]. Comprehensive reviews further confirm positive associations between both gross and fine motor proficiency and executive functioning across childhood and adolescence [4]. Beyond cognition, the acquisition of motor skills supports perceptual development and social–emotional well-being [5].

Developmental Coordination Disorder (DCD) is defined in the DSM-5 as a marked impairment in acquiring and executing coordinated motor skills, with performance substantially below age expectations [6]. These motor deficits interfere with academic tasks, daily living activities, and participation in recreational pursuits [7,8]. Prevalence estimates of DCD range from 5% to 6% among children age 5–11 worldwide, with boys more frequently affected than girls [9]. Comorbidities with ADHD, developmental dyslexia, and autism spectrum disorders are common, complicating both diagnosis and intervention planning [10].

Neuroimaging and behavioral studies indicate atypical activation in neural networks of planning and attentional regulation among children with motor coordination difficulties [11]. Correspondingly, impairments in inhibition, working memory, and strategic planning are frequently observed and closely linked to academic underperformance and everyday challenges [12,13].

Experimental paradigms reveal that dual-task conditions—such as combining gross motor activity with working memory demands—amplify the motor–executive relationship, whereas passive or low-load tasks yield weaker associations. This underscores the importance of task complexity and concurrent processing demands in shaping motor–cognition links [11,13]. Overall, prior studies indicate that the demands of tasks can either enhance or diminish the relationship between motor performance and EF. For instance, dual-task conditions that combine gross motor activity with working memory or inhibition (e.g., walking while sorting objects based on rules) tend to reinforce the association between executive function and motor control [13]. In contrast, passive observational tasks or low-cognitive load activities (e.g., drawing shapes without sequencing demands) may elicit weaker EF–motor correlations. Additionally, complex visual search tasks with fine motor coordination have demonstrated varying levels of inhibitory control and attentional engagement based on stimulus complexity [11].

Prior studies have explored the relationship between executive functions and motor performance, suggesting that deficits in attention and visuospatial integration may co-occur with coordination challenges [4,13]. Although much research has focused on clinically diagnosed DCD, fewer studies have examined how subthreshold motor coordination difficulties manifest in educational settings and relate to neuropsychological profiles. To address this gap, the present study adopts the PASS theory of information processing—a neurocognitive framework deriving from Luria’s work—to investigate interrelated domains (planning, attention, and simultaneous processing) in children with observable motor coordination challenges [14,15].

### 1.2. The Neurocognitive PASS Theory

The PASS (planning, attention, simultaneous, and successive) theory of cognitive processing, developed by Naglieri and Das [14,15], offers a robust framework for understanding human intellect. This theory is rooted in the foundational work of Russian neuropsychologist Alexander Luria, who posited that cognitive functions are managed by three interconnected functional units of the brain. Within this model, Luria identified four fundamental neurocognitive mechanisms: planning, attention, simultaneous processing, and successive processing. These mechanisms, while distinct, operate in an integrated manner to facilitate complex cognitive abilities. Recent confirmatory factor analysis of the CAS-2 standardization sample provides robust support for this four-factor correlated model [16]. This paper specifically focuses on the PASS neurocognitive processes of attention, planning, and simultaneous processing—critical for visuomotor integration—and explores how these processes function and interact in children with motor coordination challenges.

This paper explores the neurobiological underpinnings of key cognitive functions within the context of brain functional units. Cortical arousal, or attention (Luria’s 1st functional unit), is primarily associated with the brain stem and reticular activating system, responsible for cortical activation. This unit interacts with the default mode network and activates both ventral and dorsal attention networks, further connecting to the frontoparietal system to support simultaneous and successive cognitive processes. The second functional unit is dedicated to information processing, with its neural correlates in the parietal, temporal, and occipital lobes. Finally, the third functional unit, governed by the frontal lobes, is crucial for goal-oriented behavior, encompassing planning, monitoring, and adaptation [17].

**Attention** is a crucial cognitive function defined by the ability to engage in focused, selective, sustained, and effortful activities while effectively resisting distractions. This capacity is closely linked to the brain stem and various subcortical structures, demonstrating reciprocal communication with higher-order executive functions (the third functional unit). Complementing attention, the ability to process information simultaneously allows individuals to synthesize disparate stimuli into interconnected groups or a cohesive whole. This is particularly evident in tasks demanding strong visuospatial and verbal–spatial skills, neural correlations of which are found in the occipital–parietal and temporal–parietal regions of the brain. These constructions are supported by neuropsychological research linking attentional control to brainstem and subcortical activation [18,19] and simultaneous processing to occipital–parietal and temporal–parietal networks involved in spatial synthesis and verbal integration [17,20].

Simultaneous processing refers to a neurocognitive skill that enables the integration of distinct stimuli into a cohesive entity or interconnected groups. This capability is essential when disparate elements need to be synthesized into a unified concept. It encompasses both visuospatial and linguistic stimuli, necessitating the use of complex grammatical structures. The spatial component of simultaneous processing focuses on the perception of stimuli and their relationships as a unified whole, often utilizing visual imagery. Meanwhile, the grammatical aspect facilitates the merging of words into coherent ideas by understanding word relationships, prepositions, and inflections, allowing individuals to derive meaning. It is crucial to acknowledge that simultaneous processing can encompass both verbal and non-verbal content. For this reason, academically, it has been related to reading and reading comprehension, whole language, verbal concepts, geometry, math, and word problems [21], as well as movement problem solving [22,23].

**Figure 1:**
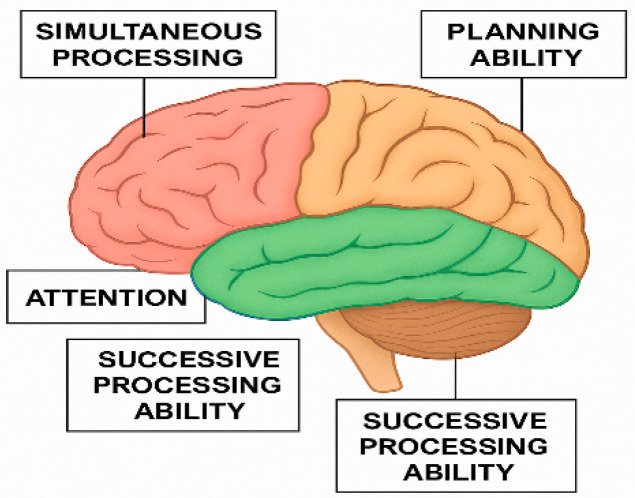
Conceptual mapping of PASS cognitive abilities to brain regions: Learning is fundamentally rooted in neurocognitive processes, specifically those categorized as PASS abilities—comprising planning, attention, simultaneous processing, and successive processing, which are primarily localized within distinct brain regions. These neurocognitive mechanisms form the foundation of learning, underpinning the acquisition of knowledge and development of skills (Adapted from [24]).

The brain’s functional units, as described by Luria [18], can now be interpreted as functional networks. These networks significantly influence various cognitive constructions, including attention, executive function, learning and memory, and information processing. Luria emphasized that cognitive activity arises from the interaction of intricate functional systems, with each system providing distinct contributions [17]. By leveraging Luria’s ideas about fundamental neurocognitive processes, Das and Naglieri created a framework to comprehend intelligence and its related capabilities αs shown in Figure 1 [24]. In line with the neurocognitive theory proposed by Das, Kirby, and Jarman, the process of acquiring knowledge is viewed as a product of the interconnected cognitive functions of planning, attention, and the simultaneous and sequential encoding and processing of information. Furthermore, all processes are shaped by the knowledge base, making the integration of knowledge crucial for achieving effective processing [25].

### 1.3. Visuospatial and Attentional Processing in Neurocognitive PASS Theory

#### 1.3.1. Visuospatial Processing

Visuospatial processing (visual/verbal spatial ability: occipital/parietal-simultaneous) plays a crucial role in human cognition, forming part of a complex and interconnected system. Essentially, it serves as one of the foundational elements that shape a person’s identity and actions [26].

Luria’s neuropsychological approach continues to be a dynamic and evolving field, particularly valuable for analyzing **spatial functions**. To gain a more precise understanding of spatial factors, neuropsychological research extends across various developmental profiles, including developmental coordination disorders. Luria’s work on the parietal lobe, for example, described **apraxia**—a form of clumsiness characterized by difficulties in executing spatially organized movements, which can manifest as constructional apraxia [18]. The association cortex, comprising the frontal, parietal, and temporal regions, is crucial for both simultaneous and sequential processing (the second functional unit). This area plays a pivotal role in higher-level cognitive functions by synthesizing information from diverse sensory and motor regions. It integrates a wide array of interconnected functions, including attention, spatial awareness, working memory, eye movements, various sensory inputs, and action coordination [20].

The parietal lobe, accounting for approximately 20% of the human brain, is critical for both sensory processing and higher-order cognition. It is primarily divided into two key areas: the somatosensory cortex, which processes tactile, proprioceptive, and nociceptive information from the body and external environment, and the posterior parietal cortex, which is involved in more complex cognitive functions and higher-level thought processes. Given its diverse roles, damage to the parietal lobe can lead to a range of significant challenges, including apraxia (difficulties with motor planning), dyscalculia (mathematical learning difficulties), and various language impairments. These issues can hinder manual tasks and lead to conditions like ‘hand alien syndrome’, along with affecting attention, memory, and movement planning. Recent studies support Luria’s findings on apraxia, particularly in pantomime. When the nondominant parietal lobe, usually the right side, is damaged, individuals may struggle with everyday skilled activities like grooming or dressing, which is referred to as apraxia. Furthermore, this damage can also disrupt their understanding of spatial relationships between objects [27]. Many of the characteristics mentioned above are recognized in the literature as being present in children diagnosed with DCD.

#### 1.3.2. Attentional Processing

**Attention** is a fundamental neurocognitive function characterized by the ability to focus on a specific stimulus while actively resisting distractions. Rooted in the brain stem, attention facilitates cortical arousal and advanced levels of engagement, essential for activating additional neurocognitive processes. For more sophisticated forms of attention, optimal arousal is crucial, particularly for “selective recognition of a particular stimulus and inhibition of responses to irrelevant stimuli” [18] (p. 271). These advanced attentional processes encompass focused and selective cognitive activities, the flexible shifting of attention based on stimulus salience, and the robust capacity to resist environmental distractions.

The development of attention encompasses both selectivity, which involves focusing on specific elements of sensory information while disregarding others, and sustained attention, which refers to the ability to remain responsive to incoming stimuli over an extended duration. Recent research highlights that the neural circuitry underlying **selective sustained attention** involves a distributed network of cortical and subcortical brain regions, with each cortical area contributing specific information-processing capabilities. Importantly, selective sustained attention established in infancy forms a critical developmental foundation for later executive function emergence. Moreover, early developing mechanisms of attentional selection, particularly those based on suppression, are vital for memory and learning processes [28]. The human attention system, evolving beyond earlier models, is now understood to comprise an alerting network (thalamic and frontoparietal regions) crucial for achieving and sustaining sensitivity to stimuli. Furthermore, two orienting networks exist: a dorsal network facilitating rapid strategic eye movements and a ventral network responsive to multimodal stimuli. Finally, two executive control networks, the frontoparietal network (for moment-to-moment task adjustments) and the cingulo-opercular network (for maintaining task goals across trials), govern higher-level attentional regulation [19].

The demand for sustained attention increases the effort required for a specific task. The Expressive Attention subtest within the CAS serves as a key illustration of how the neurocognitive process of attention is utilized. The Expressive Attention subtest asks students to recognize a specific feature of a target stimulus, such as the color blue, while ignoring distractions, like the word “red” printed in blue ink. *Focused attention* enables the identification of a particular stimulus (like a visual or audio cue); *selective attention* helps inhibit responses to distracting elements, concentrating on a task; and *sustained attention* ensures ongoing effort over an extended period, demanding long-term concentration, such as watching a screen or picture cards with multiple stimuli or preparing for an exam.

#### 1.3.3. Planning and Decision-Making Processing

Planning directly maps onto Luria’s third functional unit, primarily associated with the frontal lobes. This is responsible for the programming, regulation, organizing plans, decision-making, and verification of activity. This unit is crucial for consciousness, impulse and self-control, goal-directed behavior, encompassing the formation of intentions, the development of plans and metacognitive strategies to achieve goals (strategic thinking), the execution of these plans, and the monitoring of performance for necessary adjustments (the ability to adjust strategies as needed based on feedback and changing circumstances).

Within PASS theory’s framework, it represents a higher-order cognitive process that enables individuals to determine, select, apply, and evaluate problem-solving strategies, especially in novel situations. This involves proactive thought, self-monitoring, and the ability to adapt to changing demands, distinguishing it as a key executive function critical for adaptive behavior and learning [25,29].

### 1.4. Study Aims and Research Questions

The primary aim of this study was to examine the neuropsychological processing profiles of preschool-aged children with clinically diagnosed Developmental Coordination Disorder (DCD) in comparison to typically developing peers. Specifically, the study focused on evaluating performance across three major domains of the Cognitive Assessment System (CAS): attention, simultaneous processing, and planning. In parallel, motor skill proficiency was assessed using the Movement Assessment Battery for Children–Second Edition (MABC-2).

The study investigated whether difficulties in visuospatial reasoning, executive planning, and attentional control co-occur with motor impairments in preschoolers diagnosed with DCD. The broader objective was to identify cognitive–motor inter-relations to inform strengths-based intervention frameworks and support early developmental monitoring.

**Research Questions**

1. Do preschoolers with DCD exhibit significantly lower scores in attention, simultaneous processing, and planning compared to typically developing peers?
2. Are specific subcomponents—such as receptive attention or figure memory—able to accurately distinguish children with DCD from those without?
3. What is the nature and strength of the relationship between motor skill proficiency (manual dexterity, ball skills, balance) and cognitive functions, as measured by the CAS?

**Hypotheses**

**H1.**  *Children with DCD will score significantly lower in attention, particularly in receptive and expressive attention, compared to controls*.

**H2.**  *Children with DCD will display deficits in simultaneous processing, especially in figure memory and verbal–spatial reasoning*.

**H3.**  *Planning abilities will be significantly reduced in children with DCD, as measured by Planned Codes and Planned Connections*.

**H4.**  *Cognitive processing scores will exhibit significant negative correlations with motor performance across MABC-2 domains*.

*Attention*, a neurocognitive process involving selective focus and resistance to distractions, is assessed through the Cognitive Assessment System (CAS) subtests of Expressive Attention, Number Detection, and Receptive Attention, evaluating concentration and distraction minimization. *Simultaneous processing*, defined as the synthesis of distinct stimuli into a unified whole, is measured by CAS subtests including Non-Verbal Matrices, Verbal–Spatial Relations, and Figure Memory, focusing on comprehending object relationships and spatial integration. Furthermore, *planning*, a form of goal-oriented thinking, is examined using CAS subtests such as Matching Numbers, Planned Codes, and Planned Connections. These cognitive abilities are intricately linked, with their effectiveness deeply influenced by an individual’s knowledge base, a fundamental component of the PASS theory that underpins all cognitive, motor, and socio-emotional functions [14,15].

In the cognitive–motor domain, it is crucial to develop and improve working memory, perception, selective attention, sustained attention, processing speed, planning, decision-making, problem-solving, and metacognition through both gross and fine motor activities that involve cognitive load. Evaluating working memory during motor tasks that impose cognitive demands necessitates the use of both straightforward and intricate subtests. These evaluations take into account multiple elements, such as attention, time, and processing speed. Based on the aforementioned reasons, the standardized tests selected for this study were the MABC-2 and CAS.

## 2. Materials and Methods

### 2.1. Participants and Selection Procedure

This study investigated cognitive profiles in 88 Greek preschoolers, equally divided into 44 children clinically diagnosed with Developmental Coordination Disorder (DCD) and 44 typically developing controls, all aged 6 years. Children in the DCD group were clinically diagnosed by licensed developmental pediatricians and occupational therapists, based on DSM-5 criteria [6]. Diagnosis confirmation was provided through parental reports and documentation from pediatric rehabilitation centers, where children were actively attending structured occupational therapy and motor learning programs. All DCD participants met the following criteria: A (significant motor deficits via Movement Assessment Battery for Children–Second Edition (MABC-2) [30]; B (motor impairment affecting daily life reported by parents); C (childhood onset); and D (no other medical or neurological conditions). No diagnostic procedures were performed by the research team or educators.

Children in the control group were matched individually to the DCD participants based on age, sex, nationality, and kindergarten location across the Attica region, using a stratified randomization protocol [31]. All control participants attended the same kindergartens and had no known history of neurodevelopmental disorders or therapeutic interventions.

To ensure comparable baseline cognitive ability across groups, the Non-Verbal Matrices subtest of the Cognitive Assessment System (CAS) [32], similar to Raven’s Colored Progressive Matrices [33], was administered as a culture-free measure of reasoning. Results confirmed that participants in both the DCD (M = 11.52, SD = 2) and non-DCD (M = 13.5, SD = 2.32) groups fell within the normal to high IQ range, ensuring that group differences were not attributable to variations in general intellectual ability.

Both assessment instruments used in this study are standardized and psychometrically validated. The Movement Assessment Battery for Children–Second Edition (MABC-2) demonstrates strong internal consistency and test–retest reliability across motor domains, with Cronbach’s alpha ranging from 0.75 to 0.89 [30]. The Cognitive Assessment System (CAS), based on PASS theory (planning, attention, simultaneous, successive), shows robust construct validity and internal reliability, with subtest reliability coefficients between 0.80 and 0.90. The Greek adaptation of the Das–Naglieri CAS was culturally tailored and rigorously standardized to ensure validity, cultural relevance, and robust normative data for Greek populations. Papadopoulos et al. [34] detail the translation, back-translation, norming, and psychometric evaluation in Greek-speaking students, reporting high internal consistency and strong criterion-related validity.

Written informed consent was obtained from all participating families. Parents confirmed their child’s diagnosis (for the DCD group) and agreed to all procedures. Educators were informed only of the child’s participation in motor development programs but were not asked to apply clinical labels, ensuring clear delineation between clinical diagnosis and classroom observation.

### 2.2. Assessing Visuospatial Intelligence Through Cross-Cultural Evaluations

**Figure 2:**
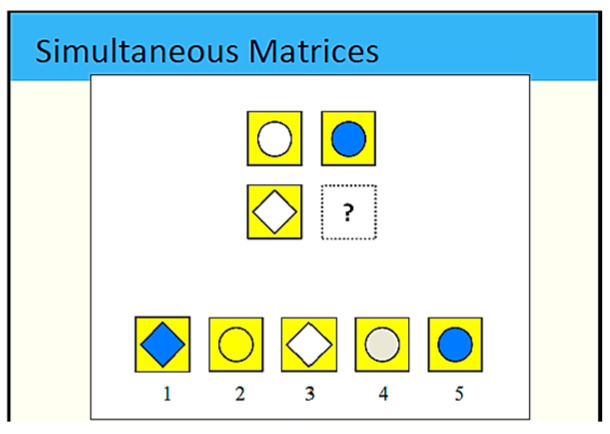
Example item from the Non-Verbal Matrices subtest.

Cross-cultural assessments of intelligence frequently utilize visuospatial intelligence tests, which involve tasks that ask participants to engage with visual representations, typically consisting of abstract geometric forms and colors. A key feature of this methodology is the visual analogy test, with Raven’s progressive matrices serving as a prominent illustration. These tests challenge individuals to discern the underlying rules linking abstract shapes organized in a matrix to identify a missing element. An example of such an item is presented in Figure 2 (Children are required to select the correct option from a set of visual choices that best completes the given pattern. This task assesses non-verbal reasoning, visual perception, and analogical thinking). 

**Figure 3:**
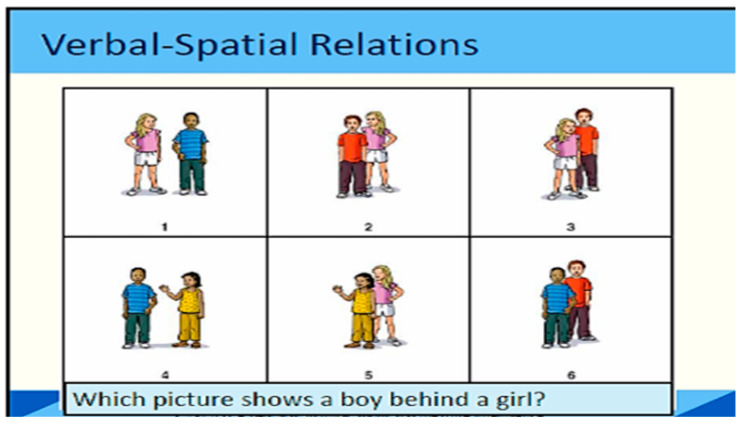
Example item from the Verbal-Spatial Relations subtest.

Raven’s Colored Progressive Matrices (RCPM) is likely the most commonly utilized in cross-cultural studies, highlighting the various cultural influences on how individuals perceive, manipulate, and conceptualize visuospatial materials. The initial step in engaging with a visuospatial test presented on paper involves recognizing the paper as a representational medium that conveys information. The paper itself is not inherently interesting; its value lies solely in the information it contains. Simply visually inspecting a test item on paper does not ensure that the test-taker will focus on it, as there may be other elements on the page that capture their attention, potentially more so than the item in question. Additionally, there are variations in how size and distance are perceived. Another pertinent consideration for intelligence assessments is whether a series of images, whether arranged in a line or a matrix, is interpreted as a sequence of states or moments in time [33]. An example of such an item is presented in Figure 3 (Children are asked to identify which image best matches a verbal description of spatial positioning—in this case, “Which picture shows a boy behind a girl?” This task assesses verbal reasoning, spatial awareness, and comprehension of relational language).

### 2.3. Measuring Instruments

#### 2.3.1. Movement Assessment Battery for Children-2, (MABC-2), (3:0–6:11 Age Band)

Movement ABC-2 (MABC-2) is a widely recognized and validated standardized assessment tool for evaluating fundamental motor skills and identifying motor impairment in children aged 3 to 16 years, encompassing both typically developing individuals and those with Developmental Coordination Disorder (DCD) [30]. This comprehensive instrument assesses a range of fine and gross motor skills, including manual dexterity, ball skills, and both static and dynamic balance, categorized across three distinct age bands. Raw scores from each task are converted into age-normed standard scores, which are then summed across all eight tasks to yield a total score and percentile rank.

These percentiles are interpreted using a “traffic light” system: scores at or below the 5th percentile (standard score ≥14) indicate significant movement difficulties, scores between the 6th and 15th percentiles (standard score ≥11 <14) suggest an increased risk, and scores above the 15th percentile (standard score ≤10) indicate no detected movement issues. All children identified as potentially at risk for DCD in the present study were evaluated using the age-appropriate MABC-2 according to its manual. The assessment of gross and fine motor skills is crucial, as these abilities are fundamental for participation in sports, recreational activities, and significantly correlate with the development of handwriting and overall academic success, including reading and math achievement in later grades [35,36].

#### 2.3.2. Cognitive Assessment System (CAS), (for Children Aged 5:0 to 18:11)

The Cognitive Assessment System (CAS) is a norm-referenced assessment tool grounded in the PASS theory of intelligence, designed to measure diverse cognitive processing abilities that may impact learning. The CAS battery helps identify specific factors such as attention, verbal and visual working memory, non-verbal ability, listening comprehension, and processing speed. For the purposes of this study, the items corresponding to the 6-year-old age group, as defined by the assessment tool, were utilized [37].

A key component of the CAS is the **Simultaneous Processing Scale**, comprising three subtests. This scale assesses a child’s capacity to identify how distinct elements integrate into a connected whole, emphasizing that despite varying content (shapes, words, numbers, musical notations) and modalities (auditory, visual); these tasks fundamentally require simultaneous processing. The three subtests are:

**Non-Verbal Matrices (33 items):** This untimed subtest presents geometric patterns and shapes, requiring children to interpret spatial or logical relationships and select one of six answers. It assesses analogical reasoning, pattern completion, and spatial visualization, with responses scored as correct (1) or incorrect (0).

**Verbal–Spatial Relations (27 items):** This subtest challenges children to understand logical and grammatical descriptions of spatial relationships. Children hear a spoken question and must match it to one of six visual illustrations within 30 s. Responses are scored as correct (1) or incorrect (0).

**Figure Memory (27 items):** In this paper-and-pencil subtest, children view a 2D or 3D shape for 5 s. After its removal, they must reproduce the exact design from memory by identifying it within a larger, more complex geometric pattern on a response sheet. Responses are scored as correct (1) or incorrect (0) based on accurate representation without additions or omissions, with no time limit for the response itself.

These simultaneous processing tasks collectively provide a comprehensive evaluation of an individual’s ability to perceive stimuli as integrated units, crucial for various cognitive functions.

**Attention Scale (3 subtests).** Child’s capacity to concentrate cognitively on specific stimuli while suppressing responses to unrelated competing stimuli is outlined (Interpretive & Technical Manual). Each subtest was specifically designed to require focused, selective, sustained, and effortful attention.

**Figure 4:**
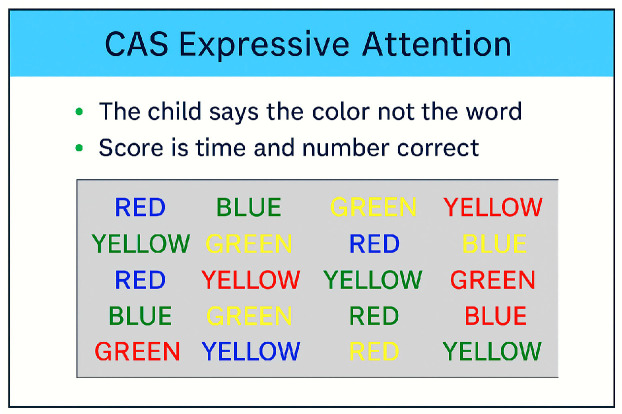
CAS Expressive Attention Subtest.

**Expressive Attention (EA)** shares characteristics with the Stroop test. In this assessment, a child is shown three sets of items, each accompanied by distinct instructions pertaining to size for younger participants and color for older ones. The sets include various distracting stimuli, all of which are connected to size or color. Each set is administered within a specific time limit. An example of such a core item is presented in Figure 4 (Sample grid from the CAS Expressive Attention subtest illustrating the color-word interference paradigm. Participants are instructed to name the color of the ink rather than read the printed word, which assesses cognitive flexibility and selective attention. It essentially measures how well a child can maintain attention and resist distraction).

**Number Detection (ND)** involves presenting a child with a page containing approximately 200 numbers displayed in various fonts. The objective is to identify and underline all instances of a designated number, or a specific number in a particular font, while disregarding distractions from the same number in different fonts or other numbers in the same font. The activity consists of four sets, along with some practice examples, and each set is timed.

**Receptive Attention (RA).** The child is shown rows of objects or letters. Each set includes a directive to underline pairs of objects or numbers, which may be based on shared names or identical sizes. There are four sets along with several practice samples, and each set is timed.

**Planning Scale (3 subtests).** The Planning Scale of the CAS specifically assesses an individual’s capacity to generate strategies, solve novel problems, and monitor their own performance. The three key subtests that assess planning are as follows:

**Planned Codes:** This subtest evaluates the examinee’s capacity to develop and apply an efficient strategy for transcribing specific letter codes based on a given legend. Successful performance hinges on systematic execution, such as completing all instances of one letter before moving to the next.

**Planned Connections:** A variation of the Trails Test, this subtest requires connecting numbers and/or letters in a specified alternating sequence (e.g., 1-A-2-B). It assesses the ability to anticipate the next item, plan the optimal path, and execute connections accurately and efficiently.

**Planned Number Matching:** This subtest presents rows of numbers, tasking children with underlining two identical numbers in each row. While seemingly straightforward, it measures planning by observing the speed, accuracy, and systematic approach used to identify matching pairs, reflecting strategic scanning.

Collectively, these subtests offer valuable insights into an individual’s capability to form intentions, formulate effective strategies, and monitor their execution within various problem-solving contexts.

#### 2.3.3. Descriptive Categories of PASS and Full-Scale Standard Scores

The score classification system for standardized assessments is based on a comprehensive full-scale standard score derived from a normative sample of 2200 participants [38]. Scores are categorized as follows: “Very Superior” for scores of 130 and above; “Superior” for 120–129; “High Average” for 110–119; “Average” for 90–109; “Low Average” for 80–89; “Below Average” for 70–79; and “Well Below Average” for scores of 69 and below. This classification provides a clear framework for interpreting an individual’s performance relative to the broader population.

### 2.4. Procedure and Ethical Considerations

This study, ethically approved by the Greek Ministry of Education, Religious Affairs and Sports, adhered fully to the principles outlined in the Declaration of Helsinki. It involved the random selection of 40 public kindergartens across Southern Greece using a stratified procedure based on region, age, and gender.

Informed written consent was obtained from all participating families. For the DCD group, parents provided confirmation of prior clinical diagnosis issued by licensed developmental pediatricians or occupational therapists, along with their child’s enrollment in afternoon motor intervention programs at affiliated rehabilitation centers. Teachers and school staff were informed about the study’s aims but were not asked to assign diagnostic labels. All data were anonymized before analysis, and no personal identifiable information was retained.

To facilitate recruitment, educators received an informational leaflet describing common characteristics of children with motor coordination difficulties, including challenges with fine motor tasks (e.g., using scissors, tying shoelaces, precise drawing), gross motor skills (e.g., balancing, throwing/catching a ball), and related behaviors (e.g., speech issues, clumsiness). This resource aimed solely to support identification for further referral and was not used to determine eligibility for the DCD group.

The study employed a matched-pairs design, in which each child with a confirmed diagnosis of DCD was paired with a typically developing child of the same age and from the same school. All neuropsychological evaluations were conducted individually by trained examiners in designated school classrooms, under standardized and controlled conditions. The assessment protocol consisted of two sessions:

- A motor skills evaluation, using the Movement Assessment Battery for Children–Second Edition (MABC-2), assessing manual dexterity, ball skills, and balance.
- A cognitive assessment, using the Das–Naglieri Cognitive Assessment System (CAS), based on the PASS theory of cognitive processing, including subtests for simultaneous processing, planning, and attention.

Each session lasted approximately 20–30 min for motor assessments and 30–40 min for cognitive testing. All assessments were carried out by the same examiner to ensure consistency across participants and settings.

### 2.5. Data Analysis

All analyses were performed in SPSS version 29.0 [39], with a predetermined significance level of *p* < 0.05. Descriptive statistics (means, standard deviations) were first computed for all dependent variables.

Prior to hypothesis testing, we verified that our continuous measures met the assumptions required for parametric procedures:

- Normality of distribution for each variable was assessed with the Shapiro–Wilk test (all *p* > 0.05).
- Homogeneity of variances between the DCD and non-DCD groups was examined via Levene’s test (all *p* > 0.05).
- Equality of covariance matrices for the multivariate analyses was checked using Box’s M test (*p* > 0.01).
- Having met these assumptions, we conducted the following steps:

i. One-Way MANOVA
  ○ We compared children with and without Developmental Coordination Disorder (DCD) across 12 motor variables (from the MABC-2),
  ○ 11 attention-related cognitive variables, and 5 simultaneous-processing cognitive variables (from the CAS PASS scales).
  ○ This analysis served two purposes: (a) to detect overall group differences on the combined set of motor and cognitive measures (Pillai’s trace); (b) to inform which variables contributed most to separating the groups.
  ○ Significant multivariate effects were followed by univariate ANOVAs on each dependent variable.

ii. Discriminant Function Analysis (Post Hoc)
  ○ We performed a stepwise discriminant function analysis to evaluate how accurately the selected motor and cognitive measures classified children into DCD versus non-DCD groups.
  ○ Structure coefficients ≥ |0.30| were interpreted as meaningful contributors to group discrimination, and overall classification accuracy was reported.

iii. Pearson’s Correlation Coefficients
  ○ Bivariate associations between the total MABC-2 score and each CAS PASS scale score were calculated across the full sample.
  ○ Correlation magnitudes were interpreted according to Cohen’s benchmarks (small: |r| = 0.10–0.29; medium: |r| = 0.30–0.49; large: |r| ≥ 0.50).

iv. Effect Sizes and Significance
  ○ For multivariate and univariate tests, partial eta-squared (ηp^2^) was reported.
  ○ Cohen’s d accompanied significant univariate contrasts.
  ○ Statistical significance was set at α = 0.05 for all tests, two-tailed.

Given the large number of dependent variables across cognitive and motor domains, we accounted for potential inflation of Type I error due to multiple comparisons. Multivariate analysis of variance (MANOVA) was employed as the primary approach to protecting against multiple testing errors. Where post hoc analyses were conducted, Bonferroni correction was applied to adjust significance thresholds and ensure robust inference. Effect sizes (η^2^) were also reported alongside *p*-values to contextualize the practical relevance of statistically significant findings.

This comprehensive strategy—grounded in appropriate assumption checks—allowed us to quantify group differences, explore the combination of measures that best distinguish DCD status, and examine the strength of motor–cognitive relationships.

## 3. Results

Descriptive statistics first established group characteristics (Table 1). Group comparisons on the Movement ABC–2 revealed that children with DCD scored substantially lower than their typically developing peers across all motor domains (Table 2). Analysis of the CAS attention subtests showed consistent deficits in the DCD group (Table 3), followed by lower performance in simultaneous processing (Table 4) and planning (Table 5). Individual profiles of children with DCD are presented in Table 6, highlighting variability across cognitive domains.

Two separate one-way MANOVAs examined group differences between children with and without DCD on (a) the 12 MABC-2 motor variables and (b) the 16 CAS-PASS cognitive measures (11 attention-related and 5 simultaneous processing). A one-way MANOVA revealed significant multivariate differences between the DCD and non-DCD groups on the attention and simultaneous PASS scales (Table 7), with results showing:

- **Attention:** Wilks’ Λ = 0.557, F(13, 74) = 4.534, *p* < 0.001
- **Simultaneous processing:** Wilks’ Λ = 0.620, F(7, 80) = 7.009, *p* < 0.001

Follow-up discriminant function analyses—rather than simple post hoc ANOVAs—identified the receptive attention total score and the simultaneous processing total score as the strongest predictors of group membership (Table 8 and Table 9), correctly classifying 73.9% and 79.5% of cases, respectively. These findings suggest that children with DCD exhibit distinct cognitive profiles, particularly in domains related to attention and simultaneous processing, which may contribute to their motor difficulties. Post hoc ANOVAs confirmed significant group differences across key variables (Table 10), and correlation analyses demonstrated robust associations between motor and cognitive domains (Table 11).

Group comparisons on the Movement ABC–2 revealed that children with DCD scored substantially lower than their typically developing peers across all motor domains. As shown in Table 2, the DCD group’s manual dexterity total (M = 7.83, SD = 2.75) was markedly higher (indicating poorer performance) than the non-DCD group’s (M = 2.12, SD = 1.88). Aiming and catching combined scores averaged M = 5.77 (SD = 2.70) for DCD versus M = 1.18 (SD = 1.20) for controls. Balance total scores followed the same pattern (DCD: M = 7.49, SD = 3.88; non-DCD: M = 1.30, SD = 1.66). Consequently, the overall total test score (TTS) was 21.10 (SD = 6.85) in the DCD group compared to 4.60 (SD = 2.93) in the non-DCD group, underscoring significant motor coordination deficits.

Analysis of the CAS attention subtests revealed that children with DCD scored consistently lower than their typically developing peers across all measures. As shown in Table 3, the DCD group’s Expressive Attention index averaged M = 27.66 (SD = 6.22) compared to M = 34.30 (SD = 6.37) in controls. Number Detection total index was M = 9.34 (SD = 2.11) versus M = 11.50 (SD = 2.27), and Receptive Attention total index was M = 7.52 (SD = 2.25) versus M = 9.70 (SD = 2.61). The overall attention PASS scaled score further highlighted this gap (DCD: M = 95.27, SD = 12.77; non-DCD: M = 109.00, SD = 13.27).

Analysis of the CAS simultaneous processing subtests indicated that the DCD group exhibited lower performance across all measures. As shown in Table 4, the non-verbal matrices index for children with DCD averaged M = 11.52 (SD = 2.08), compared to M = 13.40 (SD = 2.32) in the non-DCD group. Verbal–spatial relations scores were M = 9.68 (SD = 3.18) for DCD versus M = 11.97 (SD = 2.59) for controls, and figure memory yielded M = 8.81 (SD = 2.01) versus M = 11.95 (SD = 2.94). Consequently, the total simultaneous processing index was M = 30.02 (SD = 4.50) for DCD and M = 37.11 (SD = 5.91) for non-DCD participants, corresponding to scaled scores of M = 99.88 (SD = 9.51) and M = 114.52 (SD = 12.32), respectively.

Analysis of the CAS planning processing subtests indicated that the DCD group exhibited lower performance across all planning measures. As shown in Table 5, the Matching Numbers index for children with DCD averaged M = 7.04 (SD = 2.20), compared to M = 10.20 (SD = 2.70) in the non-DCD group. Planned Codes scores were M = 8.61 (SD = 2.32) for DCD versus M = 11.98 (SD = 2.84) for controls, and Planned Connections yielded M = 5.59 (SD = 3.64) versus M = 9.90 (SD = 3.19). Consequently, the total planning processing index was M = 21.25 (SD = 6.77) for DCD and M = 32.11 (SD = 7.58) for non-DCD participants, corresponding to scaled scores of M = 81.79 (SD = 14.00) and M = 104.34 (SD = 15.80), respectively.

In this study, the analysis of the “traffic light” system and the results of the total test score (TTS) of the MABC-2 indicated that 39.7% of the overall sample was categorized in the red zone (comprising 35 preschoolers with DCD), signifying considerable movement challenges. Furthermore, 10.3% of the participants fell into the amber zone (comprising 9 preschoolers with DCD), while 50% were classified in the green zone (44 preschoolers without DCD).

For the DCD group, the descriptive categories of PASS and full-scale standard scores [37] for attention revealed the following distribution: 1 participant was well below average, 4 were below average, 12 were low average, 24 were average, 2 were high average, and 1 was superior.

In terms of simultaneous processing for the DCD group, the scores were as follows: 1 participant was well below average, 4 were low average, 33 were average, 5 were high average, and 1 was superior.

For the non-DCD group, the descriptive categories of PASS and full-scale standard scores for attention showed that 1 participant was low average, 22 were average, 12 were high average, 7 were superior, and 2 were very superior.

Regarding the non-DCD group and simultaneous processing, the scores were as follows: 1 participant was low average, 14 were average, 16 were high average, 9 were superior, and 4 were very superior.

Analysis of individual profiles for children with DCD highlights variability not only in motor performance but also across cognitive domains. In Table 6, we list each child’s total test score (TTS) on the motor assessment, alongside their scaled scores on the three CAS cognitive scales.

Table 6 revealed that children with DCD may exhibit challenges in attention, simultaneous processing, planning, and decision-making separately or in combination, while some may not show any difficulties in these areas, regardless of the severity of their movement challenges (severe or moderate). Those with scores categorized as “Average” (100) “Borderline” (90–99) suggests performance that is slightly lower than average but not significantly so; “Low Average” (80–89), “Below Average” (70–79), and “Well Below Average” (69 and below) may be characterized as having comorbid attention disorders. Furthermore, these children tend to experience greater difficulties with attention compared to simultaneous processing.

The findings from the multivariate analysis of variance (MANOVA) concerning the attention items of the CAS revealed notable differences between the two groups of children. As illustrated in Table 7, the scores indicate that children with DCD performed significantly lower than their non-DCD counterparts.

The discriminant function analysis showed that the standardized composite score (converted from raw scores) on the Receptive Attention scale effectively differentiated between children with and without DCD. Overall, 73.9% of the children were accurately classified into the DCD and non-DCD groups. All indicators reached statistical significance (see Table 8).

The discriminant function analysis revealed that the “simultaneous processing total score anagogy index” effectively distinguished between children with and without DCD. Overall, 79.5% of the children were accurately classified into the DCD and non-DCD categories. All indicators were found to be significant (refer to Table 9).

MANOVA analysis revealed significant differences between the two groups exclusively in the domain of simultaneous processing items of the CAS (refer to Table 10).

This study conducted comprehensive statistical analyses to explore differences in motor and cognitive abilities between groups, including children with Developmental Coordination Disorder (DCD). Univariate analyses of variance (ANOVAs) revealed significant differences across all 12 motor, 11 attention, and 5 simultaneous processing standardized scores (*p* < 0.001), with post hoc ANOVAs indicating that the DCD group scored significantly lower on all subtests, highlighting substantial difficulties in both domains. Complementary *t*-tests further confirmed significant group differences (*p* < 0.05) in manual dexterity, ball skills, balance, attention, and simultaneous processing. While acknowledging the increased risk of Type I error with multiple univariate tests, the consistent significance reinforces these findings.

Furthermore, correlation analyses (detailed in Table 11) demonstrated that all investigated PASS cognitive processes (including three simultaneous and three attention subtests) were significantly associated with motor skills in both DCD and non-DCD children. Specifically, each cognitive subtest score and the total cognitive score for each scale exhibited significant negative correlations with every motor task and the total test score (TTS) for both groups. Finally, Pearson correlation coefficients for the MABC-2 domain subscores and the attention, simultaneous, and planning indices (presented in Table 11) confirmed statistically significant intercorrelations among these three assessment domains for both groups, reflecting their interconnectedness.

Interpretation: Across all cognitive domains assessed via the PASS model, lower motor performance was significantly associated with diminished attention, visuospatial processing, and planning abilities—particularly in children diagnosed with DCD. The strongest correlation was observed between planning deficits and total motor scores, underscoring the interconnected nature of executive function and gross/fine motor coordination.

## 4. Discussion

### 4.1. Summary of Key Findings

The results of this study supported all four hypotheses. Six-year-old children with Developmental Coordination Disorder (DCD) scored significantly lower than their typically developing peers across every PASS domain of the Cognitive Assessment System (CAS) and on the Movement Assessment Battery for Children—Second Edition (MABC-2). Specifically, the mean CAS attention scaled scores were 95.27 for the DCD group versus 109.00 for controls; simultaneous processing averaged 99.88 versus 114.52; and planning was 81.79 versus 104.34.

Discriminant analyses demonstrated that the receptive attention index and the total simultaneous processing score classified group membership with 73.9% and 79.5% accuracy, respectively, underscoring their diagnostic utility. Receptive attention and figure memory emerged as especially strong discriminators, in line with prior findings on attentional deficits among motor-impaired children [4].

Negative correlations between MABC-2 and CAS scores reinforced the tight interconnection between motor and cognitive development. Notably, planning scores in the DCD group were markedly lower, confirming hypothesis H3 and highlighting executive planning as a particularly vulnerable domain. Together, these findings suggest that joint administration of the CAS and MABC-2 can effectively identify preschoolers in need of cognitive–motor support, while the observed variability in individual profiles points to the necessity of tailoring intervention plans to each child’s unique strengths and weaknesses.

### 4.2. Task Specificity and Cognitive–Motor Interdependence

Our results extend emerging evidence that task demands modulate executive–motor correlations. Dual-task conditions—such as navigating a balance beam while processing verbal instructions—strengthen the association between executive function and motor coordination, whereas static or low-cognitive-load tasks yield weaker relations [13]. These findings support developmental models, emphasizing task specificity in both assessment and intervention. They also confirm that motor impairments in DCD rarely exist in isolation but co-occur with deficits in attentional control, strategic planning, and information synthesis [8,11].

### 4.3. Implications for Assessment and Intervention

Our data advocate for routine cognitive profiling, alongside screening in DCD. Incorporating PASS-based measures reveals individual profiles of strengths and weaknesses, guiding the design of integrated interventions. For example, children with planning deficits—who struggle to anticipate movement outcomes or adjust strategies—may benefit from planning facilitation protocols that scaffold metacognitive awareness and strategic thinking [17]. Embedding executive function exercises within motor training (e.g., balance drills under increasing cognitive load) can exploit transfer effects and maximize therapeutic gains.

### 4.4. Personalization, Emerging Technologies, and Group Approaches

The heterogeneity of cognitive–motor profiles in our sample—with 17 of 44 children showing deficits in only one or two PASS domains and 8 exhibiting no significant CAS weaknesses—underscores the inadequacy of “one-size-fits-all” models. A strengths-based framework should leverage intact processing domains (e.g., strong simultaneous processing to support attention tasks) and tailor objectives to each child’s unique profile. Emerging technology-enhanced tools—such as adaptive exergames, virtual-reality platforms, and tablet-based programs—offer multisensory, feedback-rich environments to concurrently train motor and executive functions [40,41]. Similarly, group psychomotor therapy and structured physical activity programs foster improvements in motor competence, cognitive control, emotional regulation, social engagement, and self-efficacy, making them powerful modalities for inclusive intervention [42].

### 4.5. Differentiating Developmental Coordination Disorder Through PASS Cognitive Subtests

Developmental Coordination Disorder (DCD) is increasingly recognized not only for its hallmark motor coordination impairments but also for concomitant deficits in higher-order cognitive processes. In the present investigation, 44 children meeting DSM-5 criteria for DCD were compared with 44 typically developing peers using three key subtests—receptive attention, simultaneous processing, and planning—drawn from the planning, attention, simultaneous, and successive (PASS) model. All participants were individually administered these subtests under standardized conditions, yielding standard scores (M = 100, SD = 15) that formed the basis for both group-level comparisons and detailed individual profile analyses.

On the receptive attention measure, the DCD cohort exhibited a markedly broader distribution of scores than controls. One child scored well below average (<80), sixteen scored below average (80–89), and the remaining twenty-seven (61%) ranged from the average band (90–109) to very superior (≥130). By contrast, typically developing children displayed a tighter clustering of attentional performance: 40% achieved average scores, 25% high average (110–119), 16% superior (120–129), and 7% very superior. This asymmetry underscores both a heavier lower-tail in attentional capacities among children with DCD and a pronounced normative clustering in controls.

Parallel divergences were observed on the simultaneous processing subtest. Within the DCD sample, one participant performed well below average, four were classified low average, thirty-three average, five high average, and one superior, whereas in the non-DCD group only one score was low average, fourteen average, sixteen high average, nine superior, and four very superior. These findings point to a significant attenuation of integrated visuospatial-assembly skills in the DCD group relative to typically developing peers.

Analysis of the planning subscale further differentiated the samples: 75% of children with DCD (*n* = 33) scored below the clinical cutoff of 90, indicating moderate to severe strategic planning deficits, whereas only 11% of controls (*n* = 6) fell below this threshold, the majority attaining scores within or above the normative range. Notwithstanding these group-level disparities, individual profile analysis revealed substantial heterogeneity among children with DCD: seventeen participants (38%) exhibited isolated deficits in one or two domains (e.g., attention and/or simultaneous processing), eight (18%) showed no impairments across the three assessed subtests, and distinct subsets demonstrated combined deficits in attention and planning or concurrent weaknesses across all three domains. These intra-group variations persisted irrespective of motor symptom severity or subtype.

To ensure diagnostic precision, a dual-criterion rule was applied such that a subtest score was deemed a clinically significant weakness only if it fell both below the individual’s PASS profile mean and beneath the standardized cutoff of 90 [43]. Employing these convergent criteria guarded against over-pathologizing and ensured that only objectively meaningful deviations informed clinical judgment. Importantly, receptive attention and simultaneous processing demonstrated robust discriminant validity, correctly classifying DCD status in approximately 74% and 80% of cases, respectively, thereby underscoring their utility as sensitive early markers.

Behaviorally, deficits in selective attention manifested as impaired filtering of irrelevant stimuli, slowed processing of visual details, increased errors from cue misinterpretation, and heightened distractibility during sustained tasks—impairments that can undermine reading comprehension, accurate information retrieval, and execution of complex visual instructions. Simultaneous processing limitations compromised the integration of component parts into cohesive wholes, while strategic planning weaknesses disrupted goal formulation, sequencing, and self-monitoring, collectively exacerbating functional inefficiencies. Considering these findings, we recommend that intervention programs be tailored to each child’s unique cognitive–motor profile, incorporating graded filtering drills to sharpen attentional selectivity, chunking strategies, and spatial-grouping techniques to bolster visuospatial synthesis, visual organizers, explicit checklists, and metacognitive prompts to scaffold planning and self-regulation. Such profile-driven remediation holds promise for ameliorating functional limitations and fostering more adaptive developmental trajectories in children with DCD.

### 4.6. Integration of Simultaneous Processing, Visuospatial Working Memory (VSWM), and Planning in Developmental Coordination Disorder: Implications for Cognitive–Motor Interventions

Simultaneous processing refers to the integrative capacity of the central nervous system to bind discrete informational elements into a coherent gestalt. When this ability is compromised, individuals manifest a reduced facility for perceiving overarching structures and for appreciating how constituent components inter-relate to form unified concepts or patterns [22]. Empirically, such impairments emerge across multiple cognitive domains. Visuospatial tasks—ranging from map interpretation and diagram analysis to complex puzzle assembly and geometric reasoning—become disproportionately challenging. In reading comprehension, an inability to apprehend thematic unity or extract principal ideas undermines processing of abstract concepts. Mathematical reasoning likewise suffers, particularly in word-problem resolution, numerical pattern recognition, and execution of multi-step solution sequences [44]. Simultaneous social cues—such as facial expressions, vocal tone, and contextual signals—may be misinterpreted or overlooked, impairing social cognition. Finally, general problem solving deteriorates when information cannot be efficiently organized into a global framework. Recognizing these manifestations is thus essential for the identification and remediation of simultaneous processing weaknesses [43].

Closely allied to simultaneous processing is the interplay between visuospatial working memory and planning. Visuospatial memory underpins spatial reasoning, wayfinding, and the coordinated execution of motor tasks—domains intrinsically entwined with planning functions. Navigation demands the recall of routes and landmarks, reliance on spatial relationships, and anticipatory mental rehearsal of turns and decision points [45]. Puzzle assembly, furniture construction, and athletic maneuvers similarly require mental manipulation and rotation of objects or body segments in space. In each scenario, effective planning presupposes the capacity to visualize successive stages, maintain spatial configurations in working memory, and update internal representations in light of ongoing sensory feedback [46].

Visuospatial working memory (VSWM) refers to the ability to temporarily hold and manipulate visual and spatial information. It allows us to remember what things look like and where they are located, even when they are no longer in view.

- **Visual component**: remembering shapes, colors, and images
- **Spatial component**: tracking positions, directions, and movement in space
- **Combined use**: mentally rotating objects, solving puzzles, navigating environments.

It plays a key role in everyday tasks like reading, writing, drawing, and moving through space. In children, strong VSWM supports academic skills such as math, spelling, and handwriting. Deficits in this area are often seen in conditions like DCD, ADHD, and dyslexia [47].

Neuroimaging investigations have converged on a frontoparietal–occipital network—encompassing the dorsolateral prefrontal cortex, superior parietal lobule, and visual association cortices—that mediates this dynamic integration of memory and executive planning [46,48]. Dual-task paradigms further corroborate the critical role of visuospatial working memory in tasks demanding concurrent attentional and navigational processing, thereby illustrating the capacity-limited nature of attentional resources when managing simultaneous information streams [48].

In the present study, the inter-relationship among attention, simultaneous processing, and planning was examined in relation to both gross and fine motor proficiency in preschoollers with DCD versus typically developing peers. Participants were assessed using the planning, attention, simultaneous, and successive (PASS) framework, alongside the Movement Assessment Battery for Children–Second Edition (MABC-2) [30]. Pearson correlation analyses across the three PASS cognitive indices and eight MABC-2 motor factors, including the total test score, yielded statistically significant associations in both DCD and non-DCD cohorts. These results underscore a robust cognitive–motor nexus irrespective of diagnostic status. Within the DCD group, inverse correlations emerged between executive functions—particularly visuospatial working memory, planning, and selective attention—and fundamental motor skills, suggesting that coordination deficits may partly stem from concomitant cognitive processing insufficiencies [6].

Theoretical models of motor skill acquisition posit that attention operates as a finite resource essential for encoding sensory information into internal representations that guide subsequent motor output [48]. In activities such as learning volleyball service, the ability to monitor performance, detect and classify movement errors, and update internal models through sensory feedback hinges on effective allocation of attentional and visuospatial memory resources [45]. This sensorimotor loop—constituting the dynamic interplay among perception, cognition, and action—provides a foundational scaffold for interdisciplinary fields spanning cognitive science, neuroscience, and biomedical engineering.

Our findings contribute several key insights to the literature. First, children with DCD exhibit pronounced difficulties in simultaneously processing multimodal stimuli, sustaining focused attention, and orchestrating sequential cognitive operations compared to their typically developing counterparts. The divergence in PASS-derived indices of simultaneous coding, attention, and decision-making tasks evidences a distinct profile of executive-function strengths and weaknesses, supporting a nuanced, profile-driven approach to eligibility determination [23]. Second, the observed cognitive–motor correlations highlight the necessity of assessing cognitive and motor abilities in tandem, employing second-generation instruments capable of isolating domain-specific competencies. Third, the impact of simultaneous processing deficits extends beyond academic domains such as reading, writing, and mathematics to physical education and motor learning contexts [41,44].

Contemporary research on DCD has shifted from broad assessments of global motor or cognitive proficiency to finely grained analyses of task-specific demands and underlying neural mechanisms. This evolution reflects an increasing appreciation for the heterogeneity of DCD manifestations and underscores the importance of aligning assessment and intervention strategies with precise cognitive–motor skills [41]. Consequently, evidence-based practice in special education and adapted physical education now emphasizes individualized teaching paradigms that calibrate stimuli, task complexity, and instructional scaffolding to the learner’s unique constellation of cognitive and motor capabilities.

In conclusion, simultaneous processing and visuospatial memory constitute core cognitive substrates for planning and motor coordination. Their dynamic interplay, mediated by overlapping neural networks and constrained attentional resources, informs both the etiology and remediation of movement-based difficulties in DCD. By integrating detailed cognitive profiling with targeted motor interventions, practitioners can devise more effective, personalized programs that foster optimal developmental trajectories for children facing these multifaceted challenges.

### 4.7. Continued Integration of Cognitive and Motor Domains in DCD: Processing Speed, Attention Development, and PASS-Based Interventions

#### 4.7.1. Processing Speed and Motor-Coding Demands

Children who struggle with coding processing speed frequently display confusion during motor-intensive tasks requiring rapid symbol reproduction from memory or swift identification of target items in number detection and receptive attention exercises. These performance breakdowns may stem from multiple interrelated factors:

- Difficulties in comprehending and executing multi-step instructions [33];
- Attentional control deficits, including reduced selective and sustained focus [19];
- Behavioral resistance, exemplified by impulsive refusals or oppositional responses during challenging tasks [49].

Beyond these influences, effective task execution relies heavily on the integration of spatial movement organization, tactile-kinesthetic perception, constructive praxis, and conceptual understanding of spatial relationships. Exposure to varied motor experiences further consolidates long-term spatial memory, while quasi-spatial patterns embedded in language reflect the pervasive role of spatial cognition in mental operations [45].

#### 4.7.2. Developmental Precursors: Attention and Executive Foundations

Extant theoretical frameworks posit that attentional capacities established during infancy constitute critical precursors to higher-order executive functions [28]. Specifically, selective sustained attention lays the groundwork for core executive processes—working memory, inhibitory control, and cognitive flexibility—by enabling the suppression of irrelevant stimuli and the maintenance of task goals over time. A systematic review of infant attention and memory systems highlights parallel trajectories in their maturation and shared neural substrates, suggesting that early-emerging attentional mechanisms catalyze the refinement of working memory and self-regulatory competencies [49].

#### 4.7.3. Emphasizing Process over Knowledge Acquisition

Contemporary cognitive assessment advocates for an emphasis on process-based evaluation—“thinking”—rather than static measures of acquired knowledge—“knowing.” Students often articulate procedural strategies (“I need a plan to solve this problem”) across domains—academic, cognitive–motor, and social—underscoring the primacy of metacognitive planning. The PASS model facilitates such process-oriented profiling, revealing individual strengths and weaknesses in cognitive strategies that traditional intelligence tests may obscure [17]. The CAS assessment invites exploration of the informational prerequisites for task completion and the impact of educational experiences on strategy development. Simple, low-threshold interventions, such as desk-mounted prompts (“If you are having trouble, use a plan”), have demonstrated efficacy in redirecting attentional focus and engaging metacognitive oversight [50].

#### 4.7.4. Multi-Faceted PASS-Based Intervention Framework

Drawing on the intervention guidelines articulated by Naglieri and Kryza [51], we propose an integrated approach to bolster children’s cognitive development within the PASS theoretical framework. Core recommendations include the following:

i. Strengths and Weaknesses Awareness: Facilitate metacognitive reflection by helping children identify their PASS profile peaks (e.g., simultaneous processing) and valleys (e.g., planning) to guide goal-directed self-regulation.
ii. Motivation and Persistence: Reinforce effortful engagement through incremental task challenges and positive performance feedback, fostering a growth mindset.
iii. Strategy Activation: Teach and model effective cognitive strategies—such as selective scanning protocols, pattern chunking, and mnemonic imagery—to enhance task-specific processing.
iv. Independence Through Metacognition: Cultivate self-efficacy by embedding opportunities for self-assessment, error detection, and strategic adjustment within ongoing learning activities.

### 4.8. Pedagogical Implications: Cultivating Metacognitive Learners

Despite the heterogeneity of task content (shapes, words, numbers, musical notation) and input modalities (auditory, visual), simultaneous processing tasks uniformly engage the same underlying neural processes. Consequently, educators should explicitly teach executive functions—attention, flexibility, inhibition, initiation, self-monitoring, working memory organization, planning, and emotional regulation—in both gross and fine motor contexts. Learners can leverage cognitive strengths (e.g., robust simultaneous processing) to compensate for attentional or planning vulnerabilities, fostering cross-domain transfer. For students exhibiting pronounced planning deficits, the planning facilitation method offers structured scaffolding to develop metacognitive control, integrating goal-setting prompts, strategy checklists, and reflective questioning to solidify planning skills [17].

By synchronizing assessment-driven insights with tailored instructional and therapeutic strategies, practitioners can more effectively address the multifaceted needs of children with DCD, thereby optimizing their developmental trajectories across cognitive, motor, and socio-emotional domains.

### 4.9. Implementation Strategies for Educators and Clinicians

Effective translation of research into practice demands structured implementation frameworks that bridge assessment insights with instructional design. A tiered support model—universal, targeted, and intensive—ensures all students benefit from executive-function (EF) and motor-skill interventions while allocating specialized resources to those with pronounced difficulties. At the universal level, teachers integrate EF prompts and movement breaks into daily routines [52]. Targeted support involves small-group sessions using ICT tools (e.g., adaptive apps, exergames) and guided group psychomotor therapy (GPT). Intensive interventions personalize goal setting and scaffold planning via the planning facilitation method for students with severe planning or simultaneous processing deficits [17].

#### 4.9.1. Teacher Training and Professional Development

Equipping educators to identify and address EF–motor links is paramount. Professional development programs should include:

- Comprehensive EF–Motor Science
  ○ Neurodevelopmental bases of DCD and ADHD [53];
  ○ PASS model essentials and their classroom manifestations [54].

- Diagnostic Observation Techniques
  ○ Differentiating inhibitory control lapses from defiant behavior [52];
  ○ Using curriculum-embedded tasks to profile EF strengths and weaknesses [50].

- Intervention Toolkit Mastery
  ○ ICT platforms: VR simulations, exergames, metacognitive apps [40];
  ○ GPT methods: activity adaptation, peer modeling, and self-action choice paradigms [55].

- Collaborative Coaching Cycles
  ○ In-class coaching with video-review feedback [42].
  ○ Peer-observation and reflective dialogue to refine EF-integrated pedagogies [51].

#### 4.9.2. Family and Community Partnerships

Sustainable EF–motor skill growth extends beyond the classroom. Engaging families and community stakeholders amplifies intervention impact through:

- Shared Understanding
  ○ Workshops explaining EF–motor interdependence and PASS processes [53];
  ○ Home–school communication logs highlighting strategy successes and challenges [17].

- Joint Strategy Development
  ○ Co-creating daily “planning check” routines (e.g., visual checklists by the breakfast table) [50];
  ○ Family-guided ICT use with mindfulness or metacognitive apps to reinforce self-regulation [40].

- Community Resources
  ○ Partnerships with local sports clubs for inclusive physical activity programs [56].
  ○ Collaboration with occupational and psychomotor therapists to align school and after-school activities [55].

### 4.10. From Cognitive Deficits to Functional Gains: A PASS-Based Intervention Model for Developmental Coordination Disorder in Early Childhood

The PASS model—planning, attention, simultaneous, and successive processing—is a framework for understanding cognitive functions. In children with Developmental Coordination Disorder (DCD), deficits in these areas often affect both motor skills (like running, jumping, writing) and executive functions (like organizing tasks or maintaining focus). Targeted intervention can improve these domains. Before intervention, children may struggle with visual attention, sequencing, and task execution. After intervention, gains are seen in motor coordination and cognitive control (see graphical abstract, p. 2).

Suggested Activities by PASS Domain [22,23,25]

Here are practical, engaging activities tailored to each PASS function:

1. Planning

- Obstacle Course Design: Let the child plan and set up a simple obstacle course using pillows, chairs, or cones.
- Step-by-Step Drawing: Follow visual instructions to draw a picture in stages.
- Cooking with Instructions: Prepare a simple snack (e.g., sandwich) by following a sequence of steps.

2. Attention

- “I Spy” Games: Focus on finding specific objects in a busy scene.
- Musical Freeze: Dance to music and freeze when it stops—great for sustained attention.
- Sorting Tasks: Sort objects by color, shape, or size under time constraints.

3. Simultaneous Processing

- Matching Pairs: Use memory cards or visual puzzles that require recognizing patterns.
- Story Sequencing: Arrange picture cards to tell a story.
- Visual Mazes: Navigate through mazes that require spatial awareness and integration.

4. Successive Processing

- Clapping Patterns: Repeat and extend rhythmic patterns (e.g., clap–clap–pause–clap).
- Follow-the-Leader Instructions: Perform a series of movements in order (e.g., jump, spin, touch toes).
- Phonics Games: Break down words into syllables or sounds and rebuild them.

*Why These Activities Matter*

These interventions help children:

- Strengthen executive functions like planning and attention.
- Improve motor coordination through structured movement.
- Enhance visual and auditory processing for academic readiness.
- Build confidence in task execution and problem-solving.

### 4.11. Limitations and Future Directions

Although integrated executive function–motor interventions show promise, several critical questions remain about how to optimize and personalize these approaches:

- Individualized Response Profiles
  ○ Which student characteristics predict the greatest gains from information-and-communication technology (ICT) versus gross-motor practice training (GPT)? [40]
  ○ How do comorbidities (e.g., ADHD, specific learning disorders) modulate intervention efficacy? [53]

- Longitudinal Outcomes
  ○ Are motor and executive function gains sustained into adolescence and early adulthood? [56]
  ○ Do improvements transfer to academic achievement, social–emotional well-being, and health-related quality of life? [57]

- Mechanistic Insights
  ○ Which neural plasticity markers track combined cognitive–motor training effects? [58,59]
  ○ What is the optimal dose–response relationship—frequency and intensity—of GPT and exergaming sessions? [42]

- Technology Integration
  ○ Can AI-driven adaptive platforms dynamically adjust challenge levels in real time? [40]
  ○ How might virtual-reality environments, grounded in embodied cognition, reinforce spatial–motor planning? [58]

Aligning theoretical frameworks with these evidence-based interventions empowers educators, clinicians, and families to coalesce around a shared vision: fostering robust executive functions alongside motor proficiency. A scaffolded, data-informed approach—spanning universal classroom practices, targeted ICT and GPT interventions, and intensive individualized support—promises enduring gains for children with DCD and related developmental challenges. Ongoing research into personalized predictors, longitudinal trajectories, and technological innovations will further refine best practices, ensuring every learner can navigate both cognitive demands and motor challenges with confidence and competence.

Despite the methodological rigor of our matched-pairs design and the ecological validity afforded by in-school testing, several limitations warrant attention:

First, although formal comorbid diagnoses (e.g., ADHD) were exclusionary, the absence of independent clinical verification leaves open the possibility that subclinical symptomatology influenced both motor coordination and executive function [6,53]. Future investigations should recruit larger, diagnostically confirmed cohorts, including children with co-occurring neurodevelopmental disorders, to capture the full continuum of individual differences within representative clinical samples.

Second, we did not systematically document participants’ engagement in ancillary therapies (e.g., occupational therapy, speech and language intervention) or organized sport activities. Consequently, it remains unclear which supplemental modalities most effectively ameliorate DCD-related deficits. Randomized or quasi-experimental trials that manipulate intervention exposure and monitor longitudinal outcomes are essential to elucidate the specific contributions of these therapeutic and recreational experiences.

Third, while we employed standardized PASS and MABC-2 measures under controlled yet naturalistic conditions, examiner subjectivity cannot be eliminated outside laboratory settings. By administering assessments within children’s own educational environments, we enhanced functional relevance—a trade-off that future research could address through the integration of wearable motion-capture devices and automated scoring algorithms to bolster objectivity and reliability [43].

Finally, our sample was confined to six-year-olds, restricting inferences about developmental trajectories across early childhood. Given evidence that cognitive–motor interdependencies evolve with age, longitudinal, age-stratified designs are needed to map sensitive periods for intervention and to tailor assessment and remediation protocols accordingly [49].

## 5. Conclusions

This study underscores the necessity of combining cognitive and motor assessments in early childhood to fully characterize Developmental Coordination Disorder (DCD). Preschoolers with DCD performed significantly below their typically developing peers in fundamental movement skills (MABC-2) and in executive functions—attention, visuospatial synthesis, and strategic planning—measured via PASS scales.

Discriminant analyses demonstrated that receptive attention and simultaneous processing reliably distinguish children with DCD from controls, correctly classifying group status in 74–80% of cases. These findings reinforce the diagnostic value of targeted PASS subtests and confirm the tight cooperation between motor coordination and cognitive processing in DCD.

From a neuropsychological perspective, dysfunctional interactions among Luria’s functional networks of attention, simultaneous processing, and planning—core to PASS theory—appear to disrupt motor network integration in children with DCD. This interdisciplinary framework reveals substantial heterogeneity in individual cognitive–motor profiles: nearly 40% of children with DCD exhibit deficits in one or two cognitive domains, while roughly 18% show no significant impairments across the three assessed areas.

Such variability demands personalized, strength-based intervention models. Effective educational and therapeutic programs should be integrated:

- Explicit instruction in cognitive strategies (e.g., focused attention drills, chunking techniques).
- Structured motor activities administered under progressively increasing cognitive load.
- Innovative digital tools (virtual-reality environments, adaptive exergames) alongside group psychomotor therapy.
- Ongoing professional development and in-class coaching for educators.

Engaging families and community partners further enhances self-regulation skills and improves overall quality of life for children with DCD.

Looking ahead, future research should investigate long-term outcomes into adolescence, track neuroplasticity markers in response to combined cognitive–motor interventions and examine dose–response relationships for group psychomotor therapy (GPT) and information and communication technology (ICT) programs. By weaving together theoretical insights, rigorous empirical findings, and applied strategies, this holistic approach promises to empower children with DCD, boosting their autonomy, self-confidence, and everyday functioning.

## Figures and Tables

**Table 1:** Descriptive characteristics of the participants (N = 88).

	Age Month		Sex	
DCD	M = 71.95 SD = 1.22	Minimum = 70 Maximum = 74	Boys = 29	Girls = 15
Non-DCD	M = 74.2 SD = 1.78	Minimum = 70 Maximum = 77	Boys = 29	Girls = 15

**Table 2:** Motor skill subtest and total scores (M, SD) for DCD and non-DCD groups.

Motor Skill Subtest	Group	Mean (M)	SD	Min	Max
Manual Dexterity Task 1	DCD	1.83	1.23	0.0	4.5
	Control	0.37	0.60	0.0	2.5
Manual Dexterity Task 2	DCD	3.61	1.12	0.0	5.0
	Control	1.36	1.48	0.0	4.0
Manual Dexterity Task 3	DCD	2.38	1.85	0.0	5.0
	Control	0.38	0.65	0.0	3.0
Manual Dexterity Total	DCD	7.83	2.75	2.0	13.5
	Control	2.12	1.88	0.0	6.0
Aiming and Catching (Combined)	DCD	5.77	2.70	0.0	10.0
	Control	1.18	1.20	0.0	5.0
Balance Total (Static + Dyn.)	DCD	7.49	3.88	0.0	15.0
	Control	1.30	1.66	0.0	7.5
Total Motor Score (TTS)	DCD	21.10	6.85	10.5	34.0
	Control	4.60	2.93	0.0	9.5

Note: DCD children show consistently lower motor performance across all subtests, with significantly reduced balance and dexterity scores. TTS indicates a clear group-level disparity. SD = Standard Deviation.

**Table 3:** CAS attention subtest scores (index and scaled) for DCD and non-DCD groups.

Subtest/Score	Group	Mean (M)	SD	Min	Max
Expressive Attention—Analogy Score	DCD	29.18	10.78	7	51
	Control	38.90	10.21	16	58
Expressive Attention—Index Score	DCD	27.66	6.22	15	40
	Control	34.30	6.37	24	53
Number Detection—Total Index Score	DCD	9.34	2.11	4	14
	Control	11.50	2.27	8	17
Receptive Attention—Total Index	DCD	7.52	2.25	2	14
	Control	9.70	2.61	7	18
Attention PASS—Scaled Score	DCD	95.27	12.77	69	121
	Control	109.00	13.27	88	148

Note: Across all attention subtests, children in the DCD group scored significantly lower than their typically developing peers. The largest disparity was observed in expressive and receptive attention measures, supporting the claim that attentional deficits are prominent in DCD.

**Table 4:** CAS simultaneous processing subtest scores (index and scaled) for DCD and non-DCD groups.

Subtest/Score	Group	Mean (M)	SD	Min	Max
Non-Verbal Matrices—Index Score	DCD	11.52	2.08	5	15
	Control	13.40	2.32	8	18
Verbal–Spatial Relations—Index Score	DCD	9.68	3.18	3	14
	Control	11.97	2.59	7	17
Figure Memory—Raw Score	DCD	8.81	2.01	6	15
	Control	11.95	2.94	5	17
Simultaneous Processing—Total	DCD	30.02	4.50	15	42
	Control	37.11	5.91	25	48
Simultaneous PASS—Scaled Score	DCD	99.88	9.51	67	124
	Control	114.52	12.32	89	138

Note: Children with DCD demonstrated weaker performance across all simultaneous subtests, particularly in verbal–spatial integration and figure memory. These differences suggest reduced capacity for synthesizing visual and verbal information into coherent wholes.

**Table 5:** CAS planning processing and decision-making subtest scores (index and scaled) for DCD and non-DCD groups.

Subtest/Score	Group	Mean (M)	SD	Min	Max
Matching Numbers—Index Score	DCD	7.04	2.20	4	13
	Control	10.20	2.70	5	16
Planned Codes—Index Score	DCD	8.61	2.32	3	14
	Control	11.98	2.84	6	17
Planned Connections—Index Score	DCD	5.59	3.64	1	14
	Control	9.90	3.19	3	16
Planning Total Score	DCD	21.25	6.77	8	37
	Control	32.11	7.58	18	46
Planning PASS—Scaled Score	DCD	81.79	14.00	55	115
	Control	104.34	15.80	75	133

Note: DCD children scored substantially lower across all planning subtests, reflecting consistent challenges in executive function areas such as strategy formulation, sequencing, and task organization.

**Table 6:** Total scores, separate for each child with DCD on the total motor score (TTS) and three cognitive scales.

DCD/N = 44/ Child ID	TTS (Motor)	Attention (Scaled)	Simultaneous Coding (Scaled)	Planning (Scaled)
1.	11	102.00	100.00	**89.00**
2.	12	**84.00**	94.00	**71.00**
3.	13	104.00	106.00	106.00
4.	13	**84.00**	98.00	94.00
5.	13	**86.00**	104.00	98.00
6.	13	98.00	94.00	**85.00**
7.	13	104.00	112.00	**67.00**
8.	13	92.00	100.00	98.00
9.	14	121.00	124.00	**89.00**
10.	15	98.00	104.00	**75.00**
11.	15	108.00	116.00	**61.00**
12.	15	96.00	100.00	**75.00**
13.	16	106.00	96.00	115.00
14.	16	106.00	106.00	**75.00**
15.	17	106.00	114.00	**75.00**
16.	17	**88.00**	92.00	**77.00**
17.	18	**82.00**	**87.00**	**81.00**
18.	18	106.00	104.00	**77.00**
19.	19	108.00	108.00	**59.00**
20.	20	**84.00**	96.00	**79.00**
21.	20	92.00	100.00	104.00
22.	20	**77.00**	106.00	**87.00**
23.	20	100.00	104.00	**89.00**
24.	21	102.00	110.00	113.00
25.	21	**88.00**	102.00	**65.00**
26.	23	104.00	102.00	**69.00**
27.	25	98.00	96.00	100.00
28.	25	**77.00**	**87.00**	94.00
29.	25	**84.00**	102.00	**75.00**
30.	25	**73.00**	**67.00**	**73.00**
31.	26	**84.00**	96.00	**83.00**
32.	26	104.00	102.00	**83.00**
33.	26	98.00	104.00	**71.00**
34.	27	**86.00**	**83.00**	**55.00**
35.	27	**73.00**	**89.00**	**73.00**
36.	28	108.00	92.00	**71.00**
37.	28	117.00	106.00	92.00
38.	29	**69.00**	96.00	**87.00**
39.	31	108.00	110.00	92.00
40.	32	**82.00**	96.00	**63.00**
41.	32	**84.00**	96.00	**81.00**
42.	33	108.00	94.00	**77.00**
43.	34	115.00	104.00	**69.00**
44.	34	98.00	96.00	**87.00**

Note: Score categories are interpreted as follows: “Average” (100) and “Borderline” (90–99) indicate performance that is slightly below average but not significantly impaired. Scores in the “Low Average” (80–89), “Below Average” (70–79), and “Well Below Average” (69 and below) ranges may be associated with comorbid attention disorders. Children with DCD tend to experience greater difficulties with attention compared to simultaneous processing. Notably, consistently low scores in the Planning scale may reflect executive functioning deficits, which are often linked to attentional challenges and reduced cognitive flexibility. Bold styling is intentionally used to highlight scores below 90, which may indicate areas of concern.

**Table 7:** A MANOVA analysis was conducted to compare the attention scale scores between the DCD and non-DCD groups.

Effect	Wilks’s Lamba	*F*	Hypo *df*	Error *df*	*p*	Eta Squared
DCD/non-DCD	0.557	4.534	13.00	74.00	<0.001	0.443

**Table 8:** Discriminant function analysis applied to the nine subtests of the attention scale.

Variable	Wilks’s Lambda	*p*	Non-Stand. Coeff.	Stand. Coeff.	Struct. Coeff.
DCD/non DCD					
Receptive Attention standard score	0.765	0.000	0.165 Constant −3.046	0.485	0.773

Note: 73.9% of original grouped cases correctly classified.

**Table 9:** Discriminant function analysis applied to the five subtests of the simultaneous scale.

Variable	Wilks’s Lambda	*p*	Non-Stand. Coeff.	Stand. Coeff.	Struct. Coeff.
DCD/ non DCD					
Simultaneous processing total score anagogy index	0.685	0.000	0.189 Constant −6.350	0.561	0.692

Note: 79.5% of original grouped cases correctly classified.

**Table 10:** A MANOVA analysis was conducted to compare the simultaneous scale results between the DCD and non-DCD groups.

Effect	Wilks’s Lambda	*F*	Hypo *df*	Error *df*	*p*	Eta Squared
DCD/non DCD	0.620	7.009	7.00	80.00	<0.001	0.391

**Table 11:** Summary of correlations between motor skills and cognitive domains.

Cognitive Domain	Associated Motor Factors	Correlation Direction	Significance Level
Attention	Manual Dexterity, Balance, Aiming, and Catching	Negative	*p* < 0.01 **/*p* < 0.05 *
Simultaneous Processing	Manual Dexterity, Static/Dynamic Balance	Negative	*p* < 0.001 **
Planning and Decision-Making	All Motor Subtests + Total MABC-2 Score	Strong Negative	*p* < 0.001 **

** Correlation is significant at the 0.01 level (2-tailed). * Correlation is significant at the 0.05 level (2-tailed).

## Data Availability

The data cannot be shared because the initial informed consent ensured privacy.
